# Group III phospholipase A_2_ promotes colitis and colorectal cancer

**DOI:** 10.1038/s41598-017-12434-z

**Published:** 2017-09-25

**Authors:** Remi Murase, Yoshitaka Taketomi, Yoshimi Miki, Yasumasa Nishito, Moe Saito, Kiyoko Fukami, Kei Yamamoto, Makoto Murakami

**Affiliations:** 1 Laboratory of Microenvironmental and Metabolic Health Science, Center for Disease Biology and Integrative Medicine, Graduate School of Medicine, the University of Tokyo, 7-3-1 Hongo, Bunkyo-ku, Tokyo, 113-8655 Japan; 2 Lipid Metabolism Project, Tokyo Metropolitan Institute of Medical Science, 2-1-6 Kamikitazawa, Setagaya-ku, Tokyo, 156-8506 Japan; 3 Core Technology and Research Center, Tokyo Metropolitan Institute of Medical Science, 2-1-6 Kamikitazawa, Setagaya-ku, Tokyo, 156-8506 Japan; 4 Laboratory of Genome and Biosignal, Tokyo University of Pharmacy and Life Science, 1432-1 Horinouchi, Hachioji, 192-0392 Tokyo, Japan; 5 Faculty of Bioscience and Bioindustry, Tokushima University, 2-1 Minami-Josanjima, Tokushima, 770-8513 Japan; 6 PRIME, Japan Agency for Medical Research and Development, Chiyoda-ku, Tokyo, 100-0004 Japan; 7 AMED-CREST, Japan Agency for Medical Research and Development, Chiyoda-ku, Tokyo, 100-0004 Japan

## Abstract

Lipid mediators play pivotal roles in colorectal cancer and colitis, but only a limited member of the phospholipase A_2_ (PLA_2_) subtypes, which lie upstream of various lipid mediators, have been implicated in the positive or negative regulation of these diseases. Clinical and biochemical evidence suggests that secreted PLA_2_ group III (sPLA_2_-III) is associated with colorectal cancer, although its precise role remains obscure. Here we have found that sPLA_2_-III-null (*Pla2g3* ^−/−^) mice are highly resistant to colon carcinogenesis. Furthermore, *Pla2g3* ^−/−^ mice are less susceptible to dextran sulfate-induced colitis, implying that the amelioration of colonic inflammation by sPLA_2_-III ablation may underlie the protective effect against colon cancer. Lipidomics analysis of the colon revealed significant reduction of pro-inflammatory/pro-tumorigenic lysophosholipids as well as unusual steady-state elevation of colon-protective fatty acids and their oxygenated metabolites in *Pla2g3* ^−/−^ mice. Overall, our results establish a role of sPLA_2_-III in the promotion of colorectal inflammation and cancer, expand our understanding of the divergent roles of multiple PLA_2_ enzymes in the gastrointestinal tract, and point to sPLA_2_-III as a novel druggable target for colorectal diseases.

## Introduction

Colorectal cancer is a frequent form of malignancy and a major cause of death in the Western hemisphere. Although physiologic levels of inflammation are protective and promote tissue repair, excessive inflammation is deleterious and lies at the basis of inflammatory bowel disease (IBD) that are ultimately linked to the development of colorectal cancer^1^. When commensal bacteria breach the colonic epithelial barrier, they trigger a state of chronic inflammation, which leads to neoplastic transformation of the overlying colorectal epithelium^2,3^. Continuous production of cytokines, growth factors, matrix proteases, angiogenic factors, and reactive oxygen species promote tumorigenesis by creating a microenvironment favoring colonic epithelial proliferation, survival, and invasiveness.

Lipid mediators represent a group of bioactive molecules that have detrimental or beneficial impacts on colorectal inflammation and cancer. Clinical use of non-steroidal anti-inflammatory drugs (NSAIDs), which inhibit cyclooxygenases (COXs) and thereby block the biosynthesis of prostaglandins (PGs) from ω6 arachidonic acid (AA; C20:4), is associated with a decreased risk of colorectal cancer^4,5^. This effect of NSAIDs is attributable mainly to reduced production of PGE_2_, since genetic deletion of biosynthetic enzymes or receptors for PGE_2_ prevents colonic carcinogenesis^6–8^. Conversely, ablation of the PGD_2_ or PGI_2_ pathway accelerates colitis or colitis-associated cancer^9–11^. ω3 Polyunsaturated fatty acids (PUFAs), such as eicosapentaenoic acid (EPA; C20:5) and docosahexaenoic acid (DHA; C22:6), are protective against inflammation and cancer in general, partly through conversion to pro-resolving lipid mediators such as resolvins and protectins^12–15^. Lysophospholipid mediators, such as lysophosphatidic acid (LPA) and sphingosine-1-phosphate (S1P), have also been implicated in colorectal homeostasis and disease^16–20^. Moreover, medium- to long-chain saturated fatty acids have aggravating effects on intestinal and systemic immunological responses, while long-chain unsaturated or short-chain fatty acids counteract these processes toward homeostatic maintenance^21,22^.

Phospholipase A_2_ (PLA_2_) is a group of enzymes that hydrolyze phospholipids to liberate fatty acids and lysophospholipids, representing the first rate-limiting step in the biosynthesis of a variety of lipid mediators. The mammalian genome encodes more than 30 PLA_2_s or related enzymes, which are classified into several subfamilies^23^. Of these, group IVA cytosolic PLA_2_ (cPLA_2_α) is coupled with the production of a large pool of colorectal PGE_2_, which promotes colorectal cancer through its receptor, EP2, and prevents colitis through another receptor, EP4^7,8,24–26^. Group IIA secreted PLA_2_ (sPLA_2_-IIA), an intestinal Paneth cell-derived sPLA_2_ also known to be a genetic modifier for tumor multiplicity in mice^27^, reduces susceptibility to intestinal tumorigenesis possibly by altering the differentiation and function of intestinal stem cells, by mobilization of eicosanoids, or by other mechanisms^28^. Group X sPLA_2_ (sPLA_2_-X), a major sPLA_2_ expressed in colonic epithelial and goblet cells, releases ω3 PUFAs, thus attenuating colitis^24^ and colorectal cancer^28^. These observations in mouse models appear to corroborate the inverse correlation between the expression levels of sPLA_2_-IIA and -X and the malignancy of gastrointestinal cancers in humans^29,30^. However, the entire picture of the PLA_2_-driven lipid pathways that are positively or negatively linked to colon pathophysiology is still not fully understood.

Group III sPLA_2_ (sPLA_2_-III), an atypical sPLA_2_ with unique structural and functional features^31,32^, has been proposed as a candidate biomarker for human colon cancer^33^. Implantation of sPLA_2_-III-transfected colon cancer cells into nude mice leads to increased growth of tumor xenografts^34^. Higher expression of sPLA_2_-III in human colorectal cancer is positively correlated with a higher rate of lymph node metastasis and shorter survival^35^. Moreover, polymorphisms in the human *PLA2G3* gene are significantly associated with a higher risk of colorectal cancer^36^. In the present study, to gain further insights into the role of sPLA_2_-III in colorectal diseases, we utilized sPLA_2_-III-deficient (*Pla2g3* ^−/−^) mice^37,38^. The results we obtained suggest that sPLA_2_-III promotes colonic cancer and colitis at least partly through mobilization of pro-inflammatory/pro-tumorigenic lysophospholipids. Thus, an agent that specifically inhibits this atypical sPLA_2_ could be useful for treatment of patients with colon disorders.

## Results

### sPLA_2_-III is expressed in the colorectal epithelium

**Figure 1:**
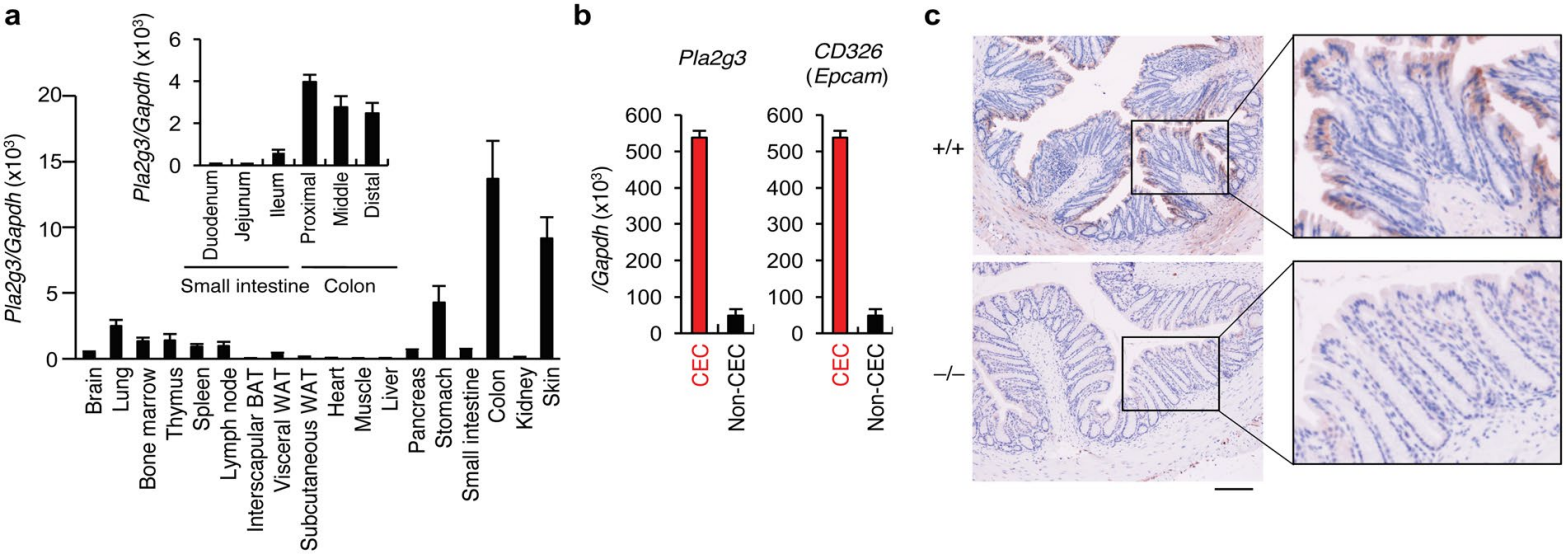
Expression of sPLA_2_-III in mouse colon. (**a**) Quantitative RT-PCR of *Pla2g3* in C57BL/6 mouse tissues (8-weeks-old males), with *Gapdh* normalization (n = 3). *Inset*, expression of *Pla2g3* in distinct portions of the small and large intestines (n = 3). BAT and WAT; brown and white adipose tissues, respectively. (**b**) Quantitative RT-PCR of *Pla2g3* and *Cd326* in CECs and non-CECs in WT colon (*n* = 3). Mean ± SEM, *P < 0.05 and **P < 0.01. (**c**) Immunohistochemistry of sPLA_2_-III in *Pla2g3* ^+/+^ and littermate *Pla2g3* ^−/−^ colons (bar, 100 μm). Boxed areas are magnified. Results are from one (**a**,**b**) and three (**c**) experiments.

Quantitative RT-PCR of C57BL/6 mouse tissues revealed high expression of *Pla2g3* mRNA in the colon and skin, followed in order by the stomach, lung, and immune organs (Fig. 1a). Within the gut tissues, *Pla2g3* was expressed throughout the proximal to distal colon, with a tendency for higher expression in the proximal than distal areas, and the expression was markedly higher than in the small intestine (duodenum, jejunum and ileum) (Fig. 1a, *Inset*). In the colon, *Pla2g3* expression was enriched in *Cd326* (*Epcam)*-positive colonic epithelial cells (CECs) (Fig. 1b). Immunohistochemistry of the wild-type (WT; *Pla2g3* ^+/+^) mouse colon showed that sPLA_2_-III protein was expressed mainly in CECs, particularly those facing the colonic lumen (Fig. 1c). No such staining was evident in the colon of *Pla2g3* ^−/−^ mice, confirming the specificity of the anti-sPLA_2_-III antibody used.

### *Pla2g3*^−/−^ mice are protected from colorectal cancer

**Figure 2:**
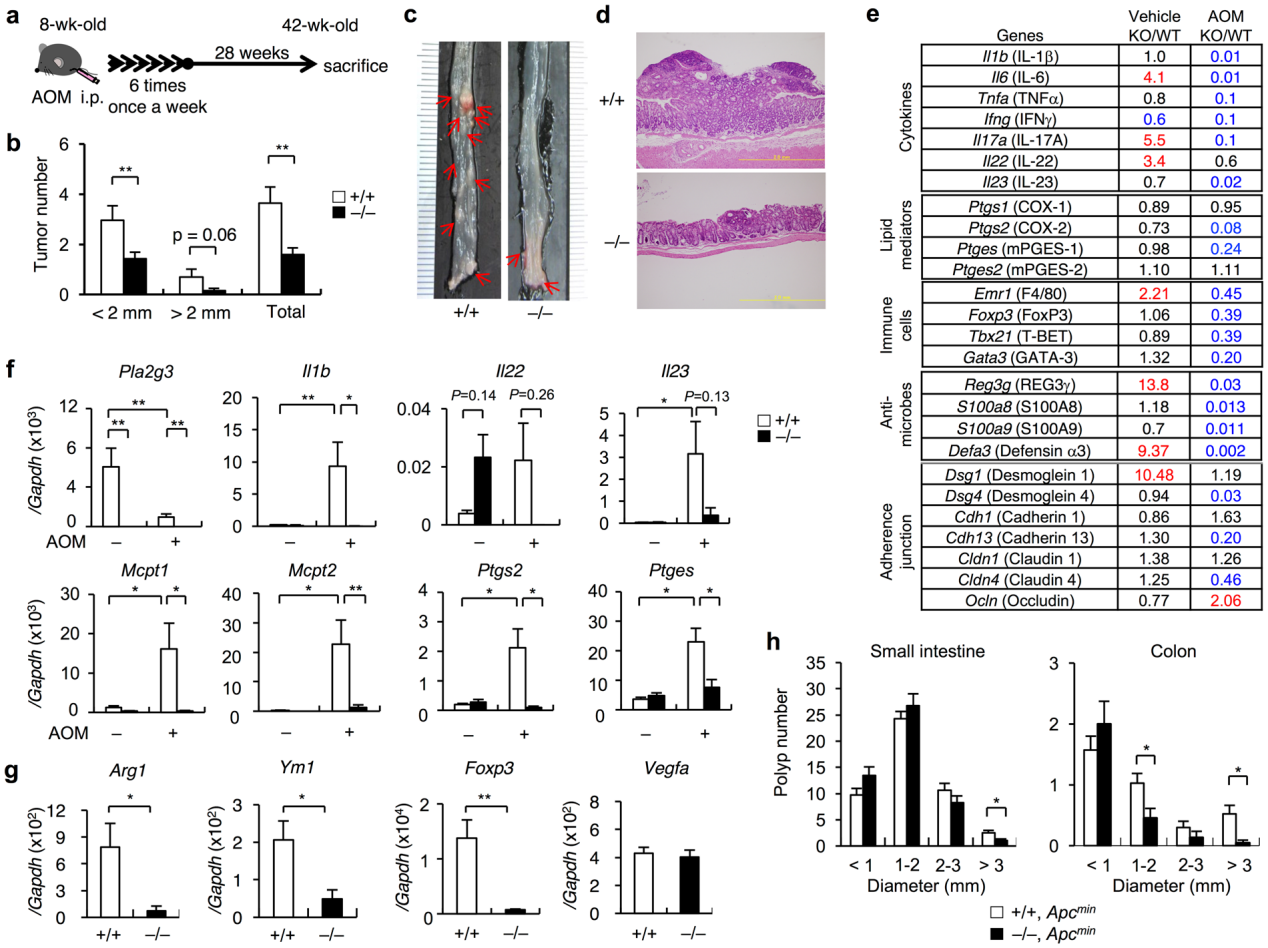
*Pla2g3* ^−/−^ mice are protected against colorectal cancer. (**a**) Schematic representation of the procedure for AOM-induced colon cancer. (**b**) Number of total, small (<2 mm in diameter) and large (>2 mm) polyps per mouse in the colons of AOM-treated *Pla2g3* ^−/−^ in comparison with littermate *Pla2g3* ^−/−^ mice (n = 19–38). (**c**) Representative photographs of colon tissues from *Pla2g3* ^+/+^ and *Pla2g3* ^−/−^ mice. Polyps are indicated by arrows. (**d**) Representative hematoxylin and eosin staining of the colon tissues from *Pla2g3* ^+/+^ and *Pla2g3* ^−/−^ mice. Bar: 200 µm. (**e**) Microarray gene profiling of the whole descending colons of *Pla2g3* ^−/−^ (KO) and littermate *Pla2g3* ^+/+^ (WT) mice with AOM or vehicle treatment. Equal amounts of total RNA (pooled from four mice for each genotype) were subjected to one-color gene expression microarray analysis. Data were processed using Agilent’s Feature Extraction Software and analyzed using GeneSpring Software. Fold changes (KO relative to WT) on the microarray are listed, and genes showing a >2-fold increase (red) or decrease (blue) in expression are highlighted. (**f**) Quantitative RT-PCR of *Pla2g3* and several inflammatory genes in the colons of *Pla2g3* ^+/+^ and *Pla2g3* ^−/−^ mice after treatment with or without AOM (*n* = 5–8). (**g**) Quantitative RT-PCR of genes for M2 macrophages, regulatory T cells, and angiogenesis in the colons of AOM-treated *Pla2g3* ^+/+^ and *Pla2g3* ^−/−^ mice (*n* = 5). (**h**) Number of polyps per mouse in the small and large intestines of *Pla2g3* ^+/+^ *Apc* ^*Min*/+^ and *Pla2g3* ^−/−^ *Apc* ^*Min*/+^ mice (4-month-old males) (n = 27–45). Mean ± SEM, *P < 0.05 and **P < 0.01. Results are from one experiment (**e**) or complied from at least two experiments (**b**,**f**–**h**).

To assess the role of sPLA_2_-III in colonic cancer, we applied a colon carcinogenesis model induced by azoxymethane (AOM), a procarcinogen that – upon metabolic activation in the liver and distal colon – induces the formation of *O* ^6^-methyl-guanine^39^, in *Pla2g3* ^−/−^ mice and also littermate *Pla2g3* ^+/+^ mice for comparison. *Pla2g3* ^+/+^ and *Pla2g3* ^−/−^ mice were intraperitoneally administered AOM once a week for 6 weeks and then sacrificed 28 weeks after the last treatment (Fig. 2a). AOM treatment induced the development of multiple tumors in the middle to distal colon of *Pla2g3* ^+/+^ mice, whereas tumor development was markedly attenuated in *Pla2g3* ^−/−^ mice (Fig. 2b–d). sPLA_2_-III deletion decreased the total tumor burden in the colon after AOM treatment, the number of large (>2 mm in diameter) and small (<2 mm) tumors being markedly lower in *Pla2g3* ^−/−^ mice than in *Pla2g3* ^+/+^ mice (Fig. 2b,c). Histologically, tumors in *Pla2g3* ^−/−^ mice were rather smaller than those in *Pla2g3* ^+/+^ mice (Fig. 2d). Thus, consistent with previous cell biological and clinical studies^33–36^, our present results lend further support to the notion that sPLA_2_-III contributes to exacerbation of colon cancer.

Microarray gene profiling of the colon revealed notable changes in the expression of a panel of genes related to epithelial homeostasis and inflammation in *Pla2g3* ^−/−^ mice, relative to *Pla2g3* ^+/+^ mice (Fig. 2e). In the vehicle-treated control group, the expression levels of *Il6*, *Il*1*7a* and *Il*2*2*, but not *Il23*, were increased in *Pla2g3* ^−/−^ mice relative to *Pla2g3* ^+/+^ mice. These cytokines regulate Th17-type immune responses, which on the one hand help to maintain the intestinal barrier, defense and repair, but can also cause immunopathology particularly in the presence of IL-23^40–45^. Consistently, expression of the IL-22-inducible anti-microbe genes *Reg3g* and *Defa3*, as well as the adherence junction protein *Dsg1*, was upregulated in the *Pla2g3* ^−/−^ colon relative to the *Pla2g3* ^+/+^ colon (Fig. 2e), suggesting enhancement of colorectal defense and barrier function in the null mice under steady-state conditions. In contrast, following AOM treatment, the expression levels of many genes related to pro-inflammatory or Th17-related cytokines (*e*.*g*. *Il1b*, *Il6*, *Tnfa*, *Ifng*, *Il17a*, *Il22* and *Il23*), lipid mediators (*e*.*g*. *Ptgs2* and *Ptges*), immune cells (*e*.*g*. *Emr1* and *Tbx21*), epithelial defense (*e*.*g*. *Reg3g*, *S100a8*, *S100a9* and *Defa3*), and adherence junctions (*e*.*g*. *Dsg4*, *Cdh13* and *Cldn4*) were reduced in the *Pla2g3* ^−/−^ colon relative to the *Pla2g3* ^+/+^ colon (Fig. 2e), implying that sPLA_2_-III deficiency protects mice from tumor-associated inflammation and homeostatic perturbation in the colon.

In support of these observations, quantitative RT-PCR revealed that the expression of inflammatory genes (*Il1b*, *Il23*, *Ptgs2* and *Ptges*) was robustly increased in the *Pla2g3* ^+/+^ colon following AOM challenge, whereas these disease-associated changes were scarcely evident in the *Pla2g3* ^−/−^ colon (Fig. 2f). The decreased induction of *Ptgs2* (encoding COX-2) and *Ptges* (encoding PGE_2_ synthase (mPGES-1)) in *Pla2g3* ^−/−^ mice relative to *Pla2g3* ^+/+^ mice implies that the tumor-associated production of PGE_2_, a pro-tumorigenic prostanoid^6–8^, is reduced by the lack of sPLA_2_-III. *Il22* expression was elevated in the basal state and decreased after AOM treatment in *Pla2g3* ^−/−^ mice relative to *Pla2g3* ^+/+^ mice (Fig. 2f), thus validating the microarray data (Fig. 2e). In line with the crucial role of sPLA_2_-III in mast cell maturation^38^, AOM-induced robust expression of mast cell markers (*Mcpt1* and *Mcpt2*) was nearly absent in *Pla2g3* ^−/−^ colon (Fig. 2f). Furthermore, in the AOM-treated group, the expression of markers for M2 macrophages (*Arg1* and *Ym1*) and regulatory T cells (*Foxp3*), which facilitate tumor growth by counteracting anti-tumor immunity^46,47^, was lower in *Pla2g3* ^−/−^ colon than in *Pla2g3* ^+/+^ colon, although that of the angiogenic marker *Vegfa* was comparable between the genotypes (Fig. 2g). Unlike the situation in human colorectal cancer^33–36^, however, colorectal *Pla2g3* expression was decreased in this colon cancer model (Fig. 2f), probably reflecting tumor heterogeneity or species difference. Nevertheless, these results collectively suggest that sPLA_2_-III promotes tumorigenesis and attendant inflammation in AOM-induced colon cancer.

To address the role of sPLA_2_-III in colon cancer further, we crossed *Pla2g3* ^−/−^ mice with *Apc* ^*Min*/+^ mice, a model of human familial adenomatous polyposis in which the oncogenic Wnt/β-catenin signal is hyperactivated due to a mutation in the *Apc* gene, leading to spontaneous development of intestinal cancer, particularly in the small intestine^48^. Although polyposis in the small intestine was barely affected by sPLA_2_-III depletion, the number of larger polyps was significantly lower in the colon of *Pla2g3* ^−/−^ *Apc* ^*Min*/+^ mice than in that of *Pla2g3* ^+/+^ *Apc* ^*Min*/+^ mice (Fig. 2h). This colon-specific effect might be attributable to the fact that sPLA_2_-III is expressed mainly in the colon, but only minimally in the small intestine (Fig. 1a). Thus, sPLA_2_-III plays an exacerbating role in colonic tumorigenesis in two distinct models.

### *Pla2g3*^−/−^ mice are less sensitive to colitis

Given that all sporadic colon cancers exhibit some aspects of inflammation and that the pathogenesis of some types of colon cancer is associated with IBD^1^, we hypothesized that the protection against colon cancer in *Pla2g3* ^−/−^ mice might be based on an ameliorated inflammatory response. To investigate this possibility, we next performed a model of acute colitis induced by dextran sulfate sodium (DSS), a sulfated polysaccharide known to be toxic to the colonic epithelium^49,50^. The DSS study is applicable to a model of IBD cancer (see below).

**Figure 3:**
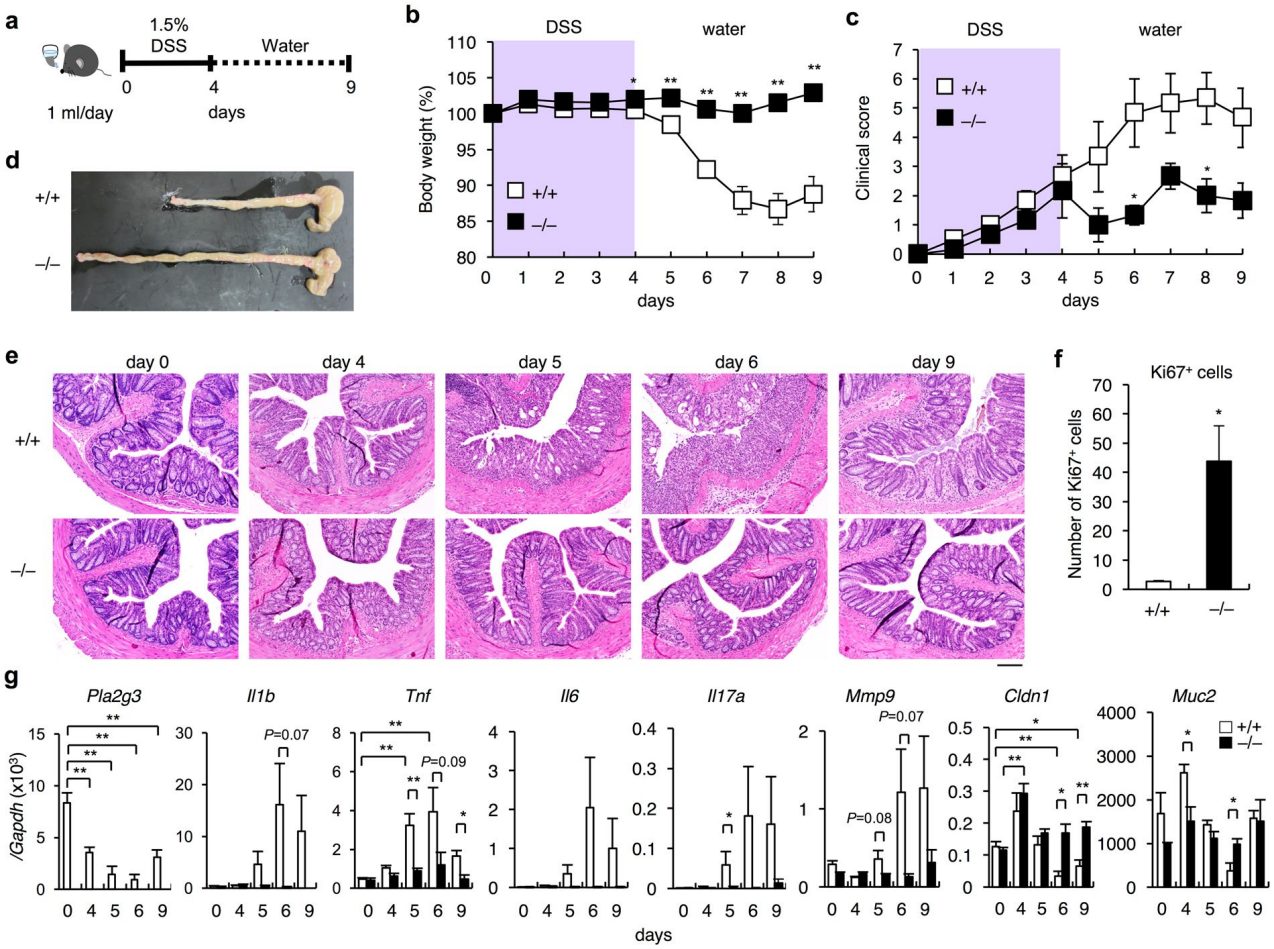
*Pla2g3* ^−/−^ mice are protected against DSS-induced colitis. (**a**) Schematic representation of the procedure for DSS-induced colitis. (**b**,**c**) Daily monitoring of body weight loss (**b**) and clinical score (**c**) in *Pla2g3* ^−/−^ and littermate *Pla2g3* ^+/+^ mice (8-week-old males) with or without DSS treatment (n = 16). (**d**,**e**) Gross appearance (**d**) and histology (**e**) of the colon in *Pla2g3* ^+/+^ and *Pla2g3* ^−/−^ mice after treatment with DSS. Bar, 100 μm. (**f**) Scoring of proliferating CEC cells per section as determined by Ki67 staining of *Pla2g3* ^+/+^ and *Pla2g3* ^−/−^ colons on day 4 (n = 3–5). (**g**) Quantitative RT-PCR of *Pla2g3* and several inflammatory or epithelial barrier genes in the colon of *Pla2g3* ^+/+^ and *Pla2g3* ^−/−^ mice after DSS treatment (*n* = 3–5). Mean ± SEM, *P < 0.05 and **P < 0.01. Data are from one experiment (**f**,**g**) or compiled from four experiments (**b**,**c**).

We administered 1.5% (w/v) DSS in water to *Pla2g3* ^−/−^ and littermate *Pla2g3* ^+/+^ mice for 4 days and then allowed the animals to recover with clean drinking water for an additional 5 days (Fig. 3a). *Pla2g3* ^+/+^ mice were susceptible to this regimen, beginning to lose body weight on day 5 and losing ~15% of their initial body weight by day 8 (Fig. 3b). This body weight change was preceded by progressive elevation of the clinical score (as monitored by fecal bleeding plus diarrhea), which began to increase on day 1 and peaked on days 6–8 (Fig. 3c). In contrast, the body weight of *Pla2g3* ^−/−^ mice remained nearly constant throughout the experimental period (Fig. 3b). Although the clinical score was gradually increased in *Pla2g3* ^−/−^ mice, it was less severe than that in *Pla2g3* ^+/+^ mice (Fig. 3c). On day 9, necropsy revealed drastic shortening of the colon length in *Pla2g3* ^+/+^ mice, whereas *Pla2g3* ^−/−^ mice were protected from this severe sign of colitis (Fig. 3d). Histologically, the *Pla2g3* ^+/+^ colon showed a progressive increase in crypt abscesses, mucosal inflammation with leukocyte infiltration, and enlargement of the muscularis propria with loss of the colonic epithelium and crypt structure by day 6, followed by apparent recovery from these symptoms by day 9 (Fig. 3e). On the other hand, these histopathological features were fairly mild in the *Pla2g3* ^−/−^ colon (Fig. 3e). On day 4, Ki67-positive epithelial cells were more numerous in the *Pla2g3* ^−/−^ colon than in the *Pla2g3* ^+/+^ colon (Fig. 3f), suggesting that the lack of sPLA_2_-III had allowed rapid recovery from epithelial injury.

In *Pla2g3* ^+/+^ mice, expression of the pro-inflammatory genes *Il1b*, *Il6*, *Tnfa*, *Ifng*, *Il17a*, and *Mmp9* was markedly induced, whereas that of the epithelial markers *Cldn1* and *Muc2*, which are crucial for epithelial barrier function^51,52^, was decreased, over days 4–9 after DSS challenge, with a peak on day 6 (Fig. 3g). In contrast, expression of these genes was affected only modestly in *Pla2g3* ^−/−^ mice (Fig. 3g). The kinetic expression profile of *Pla2g3* in the WT colon was similar to that of *Cldn1* and *Muc2*, consistent with its localization in the colorectal epithelium, which collapsed during DSS-induced injury. These results suggest that, in contrast to cPLA_2_α and sPLA_2_-X, which exert protective effects against DSS-induced colitis^24^, sPLA_2_-III has a promoting role in this disease model. Immunohistochemistry of DSS-treated WT colon revealed that, in addition to collapsing epithelial cells, some infiltrating inflammatory cells were sporadically stained with anti-sPLA_2_-III antibody (Supplementary Fig. 1). Therefore, it is possible that during colitis, sPLA_2_-III released from these particular immune cell populations might participate in the progress of the disease.

**Figure 4:**
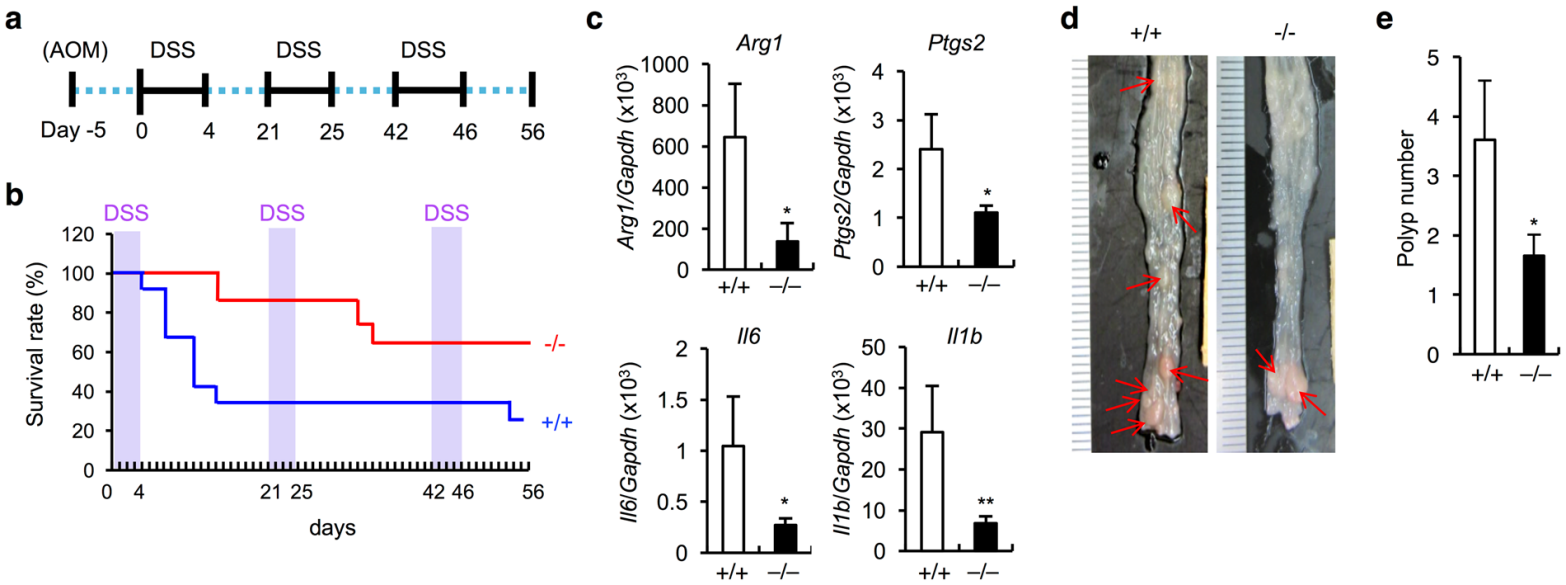
*Pla2g3* ^−/−^ mice are protected against colitis-induced colorectal cancer. (**a**) Schematic representation of the procedure for the AOM + DSS-induced colon cancer model. (**b**) Monitoring of survival rates of *Pla2g3* ^−/−^ and littermate *Pla2g3* ^+/+^ mice following repeated DSS challenges. A representative result of three experiments, starting from 12 and 14 *Pla2g3* ^+/+^ and *Pla2g3* ^−/−^ mice, respectively, is shown. (**c**) Quantitative RT-PCR of inflammation-associated genes in the colons of DSS-treated *Pla2g3* ^+/+^ and *Pla2g3* ^−/−^ mice on day 56 (n = 3 and 9 for *Pla2g3* ^+/+^ and *Pla2g3* ^−/−^ mice, respectively, in one experiment). (**d**) Gross appearance of the colons in *Pla2g3* ^+/+^ and *Pla2g3* ^−/−^ mice after treatment with AOM + DSS on day 56. (**e**) Number of large (>2 mm) polyps per mouse in the colons of AOM + DSS-treated *Pla2g3* ^+/+^ and *Pla2g3* ^−/−^ mice on day 56 (n = 23–29; compiled from two experiments). Mean ± SEM, *P < 0.05 and **P < 0.01.

To further assess the relationship between the ameliorating effect of sPLA_2_-III deficiency on colitis (Fig. 3) and that on colorectal cancer (Fig. 2), we next subjected *Pla2g3* ^−/−^ and control *Pla2g3* ^+/+^ mice to chronic DSS treatment (Fig. 4a). With this regimen, *Pla2g3* ^+/+^ mice progressively succumbed to the repeated DSS challenges, around 75% having died by the end point, whereas *Pla2g3* ^−/−^ mice were more resistant (Fig. 4b). Quantitative RT-PCR of the colon revealed markedly reduced expression of pro-inflammatory or pro-tumorigenic genes (*Arg1*, *Ptgs2*, *Il1b* and *Il6*) in *Pla2g3* ^−/−^ mice relative to *Pla2g3* ^+/+^ mice (Fig. 4c). Moreover, following the AOM + DSS regimen, in which DSS-induced chronic inflammation leads to the development of colon cancer^53^, the colon of surviving *Pla2g3* ^−/−^ mice had a lower burden of large tumors (>2 mm in diameter) than did replicate *Pla2g3* ^+/+^ mice (Fig. 4d,e). Taken together, these results indicate that the inflammatory microenvironment created by sPLA_2_-III enhances the development of tumors.

### Colonic sPLA_2_-III mobilizes lysophospholipids

**Figure 5:**
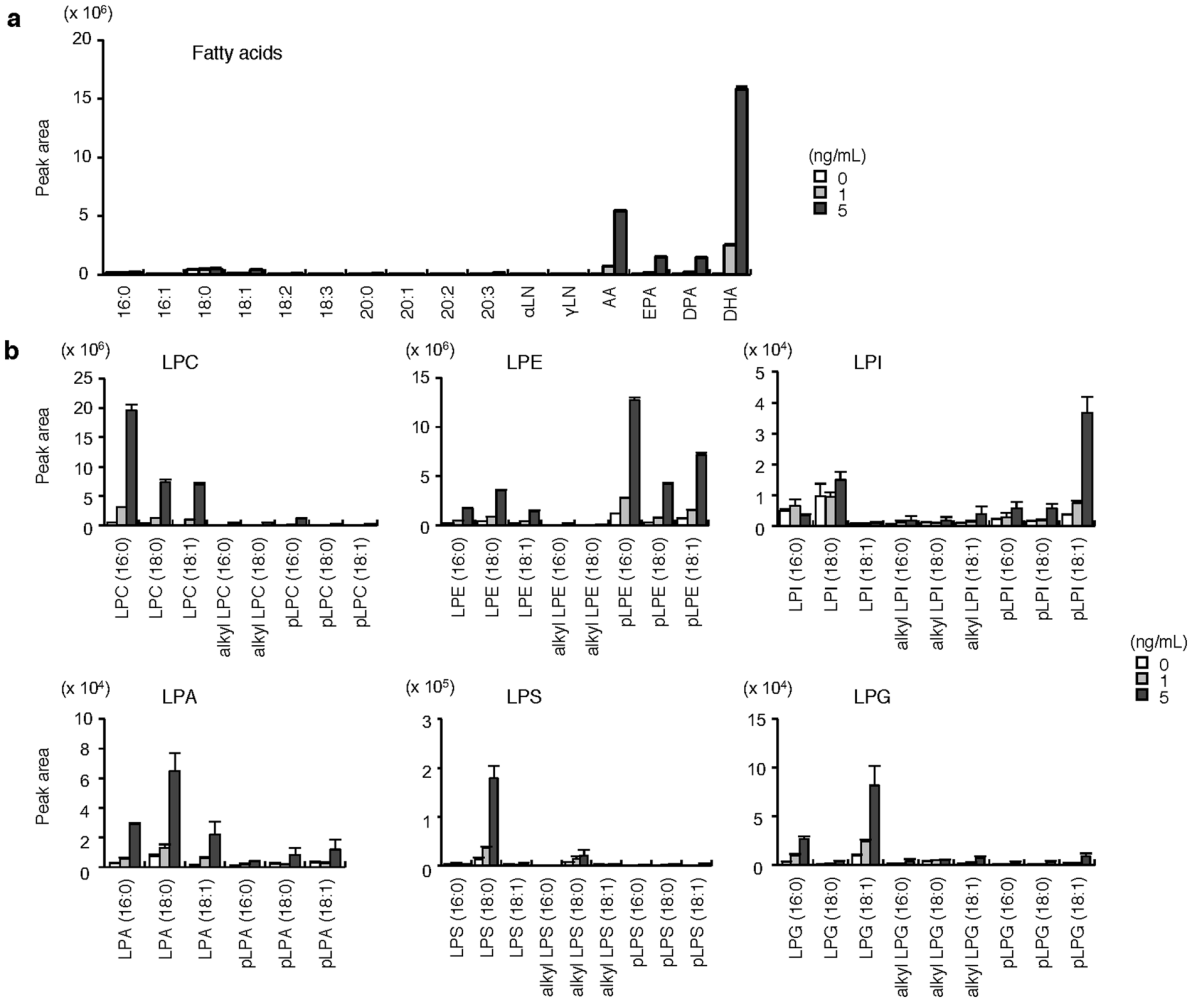
*In vitro* enzymatic activity of sPLA_2_-III on phospholipids extracted from the colon. Release of fatty acids (**a**) and lysophospholipids (**b**) from colon-extracted phospholipids (10 μM) after incubation for 30 min with the indicated concentrations of recombinant sPLA_2_-III (n = 4). Individual lysophospholipid and fatty acid species were evaluated by ESI-MS. Values are mean ± SEM. LPE, lysophosphatidylethanolamine; LPS, lysophosphatidylserine; LPG, lysophosphatidylglycerol. Representative results of two experiments are shown.

Under *in vivo* conditions, lipid mobilization by sPLA_2_ depends not only on its intrinsic substrate specificity, but also on the spatiotemporal availability or phospholipid composition of target membranes in a given tissue microenvironment, which explains why distinct sPLA_2_s exert specific functions with different lipid profiles in distinct settings^23^. For instance, colorectal sPLA_2_-X preferentially releases anti-inflammatory ω3 PUFAs, thereby exerting a protective effect against DSS-induced colitis^24^. We reasoned that the pro-inflammatory and thereby pro-tumorigenic actions of sPLA_2_-III might rely on a unique form of lipid metabolism possibly differing from that driven by sPLA_2_-X. With this possibility in mind, we reevaluated the *in vitro* enzymatic action of recombinant sPLA_2_-III on tissue-extracted natural membranes by lipidomics analysis using electrospray ionization mass spectrometry (ESI-MS)^54,55^. Incubation of bulk phospholipids extracted from mouse colon with recombinant sPLA_2_-III resulted in dose-dependent increases of AA and DHA in preference to other fatty acids (Fig. 5a), as well as increases in various lysophospholipid species bearing a saturated or monounsaturated fatty acid (Fig. 5b). These results confirmed that sPLA_2_-III has the capacity to hydrolyze phospholipids with a tendency for *sn*-2 PUFA preference without apparent polar head group selectivity *in vitro* ^23^.

**Figure 6:**
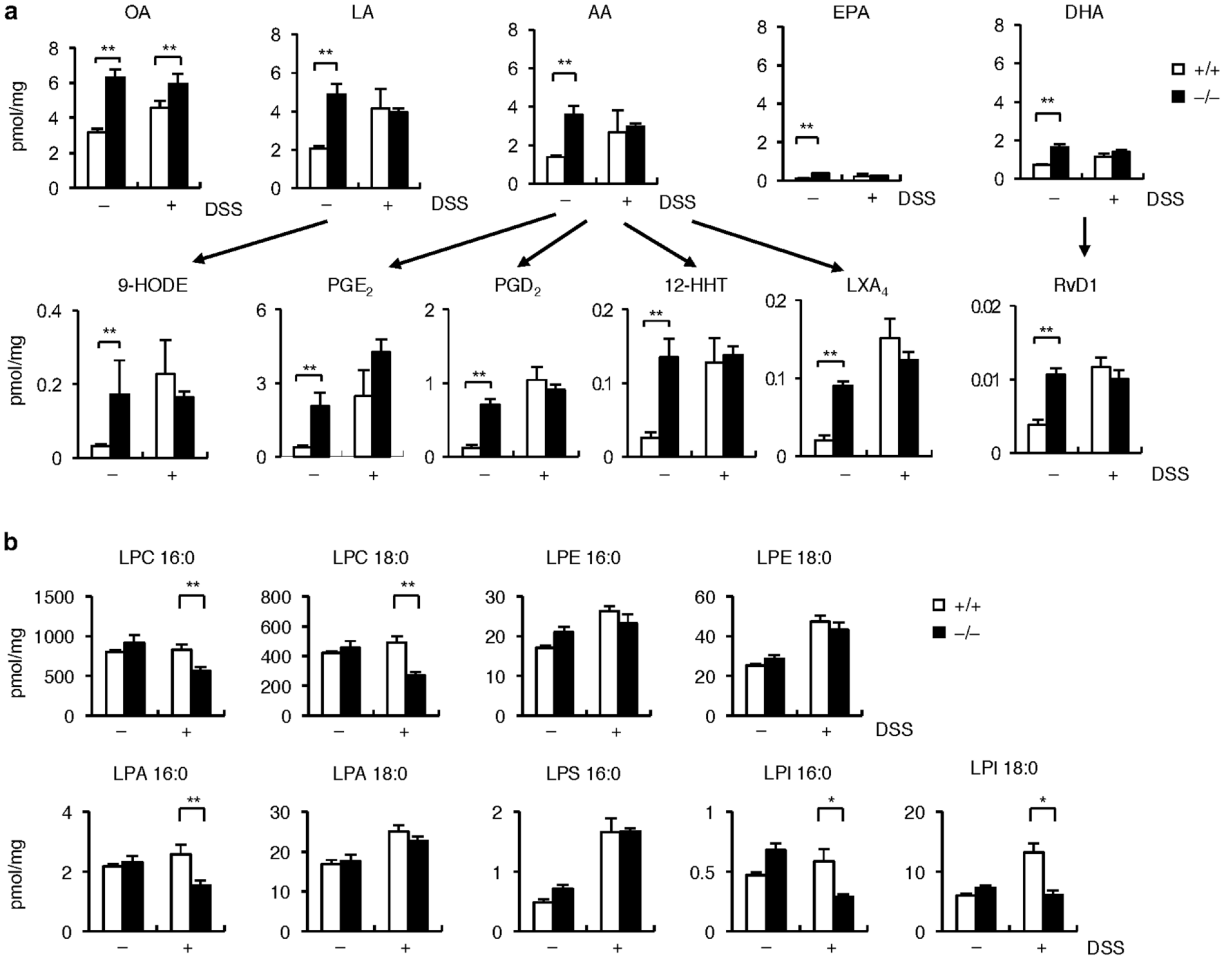
Lipidomics analysis of the colons of *Pla2g3* ^+/+^ and *Pla2g3* ^−/−^ mice. ESI-MS analysis of fatty acids and their metabolites (n = 7–8) (**a**) and lysophospholipids (n = 5–7) (**b**) in the colons of *Pla2g3* ^−/−^ and littermate *Pla2g3* ^+/+^ mice with (+) or without (−) DSS treatment for 4 days. Mean ± SEM, *P < 0.05 and **P < 0.01. Representative results of two experiments are shown.

Having established the enzymatic property of sPLA_2_-III, we next performed lipidomics analysis of the colon from *Pla2g3* ^+/+^ and *Pla2g3* ^−/−^ mice to identify particular lipid products (fatty acids, lysophospholipids and their metabolites) that were altered by sPLA_2_-III deficiency *in vivo*, under the assumption that the sPLA_2_-III-driven lipid products would be decreased in the null mice. For this purpose, we chose a condition of acute DSS-induced colitis, since chronic inflammation and cancer would be expected to be associated with global alteration in lipid metabolism (see below), making precise assessment of the sPLA_2_-III-intrinsic action difficult. In *Pla2g3* ^+/+^ mice, the colorectal levels of free fatty acids and their metabolites were elevated to various degrees following DSS treatment (Fig. 6a), as expected from our previous study^24^. However, none of these lipids were decreased in *Pla2g3* ^−/−^ colon relative to *Pla2g3* ^+/+^ colon regardless of DSS challenge; in fact, they were unexpectedly elevated in the null mice, particularly under steady-state conditions (Fig. 6a). These lipids included fatty acids [oleic acid (OA, 18:1), linoleic acid (LA, 18:2), AA, EPA and DHA] as well as their oxygenated metabolites including 9-hydroxyoctadecaenoic acid (9-HODE), PGE_2_, 12-hydroxyheptadecatrenoic acid (12-HHT), lipoxin A_4_ (LXA_4_) and resolvin D1 (RvD1), which have been reported to facilitate colorectal barrier function, defense and repair, and prevent colonic inflammation^14,26,56,57^. After DSS treatment, these differences between the genotypes were masked by overall elevation of fatty acids and their metabolites (except for OA, which was significantly greater in the null mice) (Fig. 6a).

Considering that the steady-state increase of fatty acids appeared to reflect a secondary, rather than direct, effect of sPLA_2_-III deficiency, we compared the expression of a wide array of lipid-metabolic genes in *Pla2g3* ^+/+^ and *Pla2g3* ^−/−^ mice using the microarray. However, the steady-state expression levels of most lipid-metabolic genes were not profoundly affected by the lack of sPLA_2_-III (Supplementary Table 1). A few exceptions included substantial elevation of *Lpcat2* (lysophosphatidylcholine (LPC) acyltransferase 2) and *Aloxe3* (epidermal lipoxygenase 3) in *Pla2g3* ^−/−^ mice relative to *Pla2g3* ^+/+^ mice, but these alterations could not fully account for the overall elevation of free fatty acids and their metabolites in the null mice. Therefore, we speculate that the steady-state elevation of fatty acid levels in the *Pla2g3* ^−/−^ colon might result from post-transcriptional modifications of some lipid-metabolic enzymes or from an alteration in colorectal microbiota that produce or consume fatty acids. Nevertheless, the steady-state increases of colon-protective fatty acid metabolites in *Pla2g3* ^−/−^ mice could have contributed, at least partly, to protection against colitis. As expected, the expression levels of many lipid-metabolic genes were altered in AOM-treated *Pla2g3* ^−/−^ mice compared with *Pla2g3* ^+/+^ mice (Supplementary Table 1), likely as a result of the marked attenuation of colon cancer in the null mice. For instance, the reduced expression of *Pla2g2d* corroborates the decrease of tumor-promoting M2-like macrophages (Fig. 2g)^58^, the reduced expression of *Plcg1* and *Plcg2* could explain the decreased growth factor signaling^59^, and the altered expression of several lipogenic, lipolytic and β-oxidation genes might reflect cancer-associated metabolic reprogramming^60^, in AOM-treated *Pla2g3* ^−/−^ mice relative to *Pla2g3* ^+/+^ mice.

Intriguingly, under DSS-treated (but not steady-state) conditions, the colonic levels of several, if not all, classes of lysophospholipids, including LPC, LPA, and lysophosphatidylinositol (LPI) bearing a saturated fatty acid, which are typical PLA_2_ reaction products, were significantly lower in *Pla2g3* ^−/−^ than in *Pla2g3* ^+/+^ mice (Fig. 6b). A large body of evidence suggests that these lysophospholipid species participate in the promotion of colitis or colonic cancer^16–19,61^. It is thus likely that sPLA_2_-III mobilizes a pool of these pro-inflammatory and/or pro-tumorigenic lysophospholipids from DSS-damaged, rather than intact, epithelial membranes, thus contributing to the exacerbation of colonic inflammation and subsequent progression to colonic cancer.

## Discussion

The proposed connection of sPLA_2_-III with colorectal cancer has arisen from the findings that overexpression of sPLA_2_-III in colon cancer cells enhances proliferation both in culture and in nude mice^34^, that higher expression of sPLA_2_-III is significantly correlated with more aggressive metastasis and poorer prognosis in patients with colorectal cancer^35^, and that human *PLA2G3* polymorphisms are significantly associated with a higher risk of colon cancer^36^. Our present study employing *Pla2g3* ^−/−^ mice provides compelling evidence for the exacerbating role of sPLA_2_-III in colorectal cancer. Given that *Pla2g3* ^−/−^ mice are also protected from colitis, the pro-tumorigenic function of sPLA_2_-III may rely, at least in part, on the ability of this extracellular lipolytic enzyme to propagate colorectal inflammation, which is now well recognized as a key mechanism underlying colorectal carcinogenesis^1–3^.

Currently, only a few PLA_2_s have been firmly assigned to colonic pathophysiology. cPLA_2_α, an AA-specific intracellular PLA_2_ that is activated by Ca^2+^ and phosphorylation following diverse stimuli^62,63^, mobilizes a large pool of colonic PGE_2_, which is protective against acute injury but accelerates chronic colitis and cancer, most likely by acting on distinct PGE_2_ receptors spatiotemporally expressed in different cells^6–8,24,25^. Thus, mice lacking EP4 display more severe acute colitis, whereas those lacking EP2 are protected from colonic cancer^8,26^. Genetic absence of sPLA_2_-IIA and -X in the small and large intestines improves recovery from intestinal inflammation but predisposes mice to colorectal cancer, and this trade-off effect has been proposed to involve sPLA_2_ receptor-dependent activation of cPLA_2_α and thereby production of PGE_2_^28^. Using knockout mouse lines for various sPLA_2_s, we have recently shown that sPLA_2_-X preferentially mobilizes anti-inflammatory ω3 PUFAs including DHA and EPA, which block harmful Th17-type immune responses and thereby attenuate colitis partly through the fatty acid receptor GPR120^24^. Our present results suggest that colorectal sPLA_2_-III drives another arm of the lipid pathways, namely the production of pro-inflammatory/pro-tumorigenic lysophosholipids, which may be eventually linked to the exacerbation of colonic inflammation and cancer. These findings accommodate an emerging concept that distinct PLA_2_s act on different membranes, thereby spatiotemporally mobilizing specific lipid products that have variable impacts on distinct stages of homeostasis and diseases even in the same tissue^23^.

In DSS-treated *Pla2g3* ^−/−^ colon, there are significant decreases in LPC, LPA and LPI species with a saturated fatty acid, suggesting that they are produced by sPLA_2_-III. LPA, a pluripotent lysophospholipid mediator with diverse functions, is produced either indirectly by conversion of phosphatidylcholine (PC) to LPC by PLA_2_ (or PLA_1_) and then to LPA by autotaxin (lysophospholipase D) or directly by PLA_2_ (or PLA_1_)-mediated conversion of phosphatidic acid (PA) to LPA^64^. A growing body of evidence suggests that LPA promotes cancer cell proliferation, motility and metastasis^65–68^. This view appears to be reminiscent of the potential association of sPLA_2_-III with colon cancer malignancy and metastasis in humans^35,36^. Indeed, the attenuated colonic inflammation and cancer observed in *Pla2g3* ^−/−^ mice are strikingly similar to those reported for mice lacking one of the LPA receptors, LPA_2_^17,69^. Although the *in vivo* roles of LPI, a PLA_2_-hydrolyzed product of phosphatidylinositol (PI), are less clear, genetic or pharmacological ablation of GPR55, an LPI receptor, has been reported to alleviate colitis^18,61^. Since sPLA_2_-III has the capacity to hydrolyze all classes of phospholipids without apparent polar head group specificity, we speculate that this sPLA_2_ may be accessible to certain membrane compartments rich in the precursor phospholipids (PC, PA and PI) within a particular colonic microenvironment, leading to generation of a pool of the pathogenetic lysophospholipid species that may act on specific receptors such as LPA_2_ and GPR55. Notably, lysophospholipid production by sPLA_2_-III is evident in DSS-treated, but not steady-state, colon, suggesting that sPLA_2_-III acts on labile or damaged epithelial membranes or on certain infiltrating leukocyte membranes in this disease setting.

Contrary to our prediction, the steady-state levels of unsaturated fatty acids and their metabolites (prostanoids, lipoxins and resolvins) were found to be noticeably elevated (rather than decreased) in *Pla2g3* ^−/−^ colon relative to *Pla2g3* ^+/+^ colon. Given the biological actions of these fatty acid metabolites^14,26,56,57^, this alteration could contribute to homeostatic protection against epithelial damage and excessive inflammation in *Pla2g3* ^−/−^ mice. Indeed, the steady-state increase of PGE_2_ could account for that of IL-22, an epithelial cytokine crucial for intestinal homeostasis^70^, in *Pla2g3* ^−/−^ mice, since this AA metabolite, by acting on its receptor EP4, promotes gut barrier function through driving IL-22 production by type 3 innate immune cells (ILC3s)^71^. AA-derived LXA_4_ and DHA-derived RvD1 are now well recognized as anti-inflammatory lipid mediators that sequester inflammatory responses in general^72^. Moreover, long-chain fatty acids can shape the host-microbiome interface by modulating homeostatic inflammasome signaling in CECs followed by IL-22 expression by ILC3s, which in turn promotes the secretion of mucins and antimicrobial peptides from CECs and the regeneration of colonic stem cells^42,73,74^.

The unusual increase of fatty acids and their metabolites in the *Pla2g3* ^−/−^ colon in the normal state could be explained by a compensatory mechanism involving an increase of lipid synthesis or a reduction of lipid consumption. However, our microarray analysis showed that the steady-state expression levels of a wide array of lipid-metabolic genes were largely unaffected by sPLA_2_-III deficiency. Since our present study suggests that the *Pla2g3* ^−/−^ colon shows signs of improved epithelial barrier function under steady-state conditions, it is tempting to speculate that certain homeostatic stimuli such as innate or adaptive immune responses triggered by commensal bacteria or nutrients, which exert various effects on intestinal physiology^42,75^, might post-transcriptionally modulate the activity, stability or localization of some lipid-metabolizing enzymes, leading to constitutive elevation of free fatty acid levels. Alternatively, the absence of sPLA_2_-III might alter colorectal microflora that produce or consume fatty acids. In this context, sPLA_2_-III constitutively secreted from CECs might affect the functions or populations of colonic stem cells, ILC3s or other cells, or even the microbiome, by mobilizing certain unidentified lipid metabolites or acting directly on microbial membranes, a possibility that remains to be investigated. Apart from the steady-state increase of PGE_2_, the absence of sPLA_2_-III markedly attenuates the tumor-associated induction of PGE_2_-biosynthetic enzymes (COX-2 and mPGES-1), implying the reduced biosynthesis of pro-tumorigenic PGE_2_ in the colon cancer state.

sPLA_2_-III secreted from mast cells promotes their maturation and accompanying anaphylaxis through a microenvironmental PGD_2_-mediated paracrine circuit^38^. Indeed, we have shown that the expression levels of mast cell markers are markedly lower in *Pla2g3* ^−/−^ colon than in *Pla2g3* ^+/+^ colon, confirming that the null mice have mast cell insufficiency^38^. Several lines of evidence suggest that mast cells often influence the pathology of colitis and cancer^76–78^, raising the possibility that sPLA_2_-III in mast cells may have a role in colorectal diseases. However, recent findings suggesting that mast cell-derived PGD_2_ prevents, rather than promotes, colon inflammation and cancer^10^ and that mast cells facilitate recovery, rather than injury, of the epithelium in DSS-induced colitis^77^ argue against this idea. Future analysis using mice with conditional *Pla2g3* deletion in CECs, mast cells, or even other cell types will provide further insight into the mechanistic action of sPLA_2_-III.

Lastly, given that sPLA_2_-III, an atypical sPLA_2_, is insensitive to classical sPLA_2_ inhibitors and that no protein structurally homologous to sPLA_2_-III is encoded in the human genome, a new agent that specifically inhibits this unique sPLA_2_ may be useful for the treatment of patients with IBD and colorectal cancer.

## Methods

### Mice

Heterozygous *Pla2g3* ^+/-^ mice (C57BL/6 × 129 Sv) were backcrossed to the C57BL/6 background for three generations, and then male and female heterozygotes were intercrossed to obtain *Pla2g3* ^−/−^ mice and littermate *Pla2g3* ^+/+^ mice^37,38^. Probably because of this genetic background, littermate *Pla2g3* ^+/+^ mice were more susceptible to colitis and colonic tumorigenesis than C57BL/6 mice, as the presence of the 129/Sv genetic background increases the sensitivity to these models^79–82^. *Apc* ^*Min*/+^ mice were purchased from Jackson Laboratory and crossed with *Pla2g3* ^−/−^ mice. C57BL/6 mice were obtained from SLC Japan. All mice were housed in climate-controlled (23 °C) specific pathogen-free facilities with a 12-h light-dark cycle, with free access to standard diet CE2 (CLEA Japan) and water. All procedures involving animals were approved by the Institutional Animal Care and Use Committees of the Tokyo Metropolitan Institute of Medical Science, in accordance with the Standards Relating to the Care and Management of Experimental Animals in Japan.

### Histology and immunohistochemistry

Formalin-fixed tissues were embedded in paraffin, sectioned, mounted on glass slides, deparaffinized in xylene, and rehydrated in ethanol with increasing concentrations of water. The tissue sections (4 μm thick) were incubated with 20 μg/ml proteinase K (Invitrogen) in phosphate-buffered saline (PBS) for antigen retrieval as required, incubated for 10 min with 3% (v/v) H_2_O_2_, washed 3 times with PBS for 5 min each, incubated with 5% (w/v) skim milk in PBS for 30 min, washed 3 times with PBS for 5 min each, and incubated with rabbit antibody against human sPLA_2_-III^83^ or Ki67 (Novus Biologicals) or with control antibody (Abcam) at 1:500 dilution in PBS overnight at 4 °C. The sections were then treated with a CSA system staining kit (Dako) with diaminobenzidine substrate, followed by counterstaining with hematoxylin.

### Quantitative RT-PCR

Total RNA was extracted from tissues or cells using TRIzol reagent (Invitrogen). First-strand cDNA synthesis was performed using a High Capacity cDNA Reverse Transcriptase Kit (Applied Biosystems). PCR was carried out using the TaqMan Gene Expression Assay (Applied Biosystems) on the ABI7700 Real Time PCR system (Applied Biosystems). The probe/primer sets used are listed in Supplementary Table 2.

### DSS-induced colitis

Mice (8 weeks old, male) were orally administered 1.5% (w/v) DSS (average molecular weight 36,000–50,000) (MP Biomedicals, Solon, OH) in drinking water for 4 days and then allowed to recover with free access to DSS-free drinking water for an additional 5 days. Changes in body weight were evaluated every day. To assess the severity of colitis, body weight, stool consistency, and occult blood in the stools were monitored daily^24^. Diarrhea was scored as follows: 0, normal; 2, loose stools; 4, watery diarrhea. Occult blood was scored as follows: 0, normal; 2, hemoccult positive; 4, gross bleeding. On the last day of the experiments, the colon was removed for histological and biochemical analyses.

### AOM-induced colorectal cancer

Mice (8 weeks old, male) were injected intraperitoneally with AOM (Wako) at a dose of 10 mg/kg body weight once a week for 6 weeks. Mice were sacrificed 28 weeks after the last injection of AOM. On the last day of the experiments, the colon was removed for histological and biochemical analyses.

### Colitis-associated colorectal cancer

Colitis-associated colorectal cancer was induced in mice (8 weeks old, male) by intraperitoneal injection with AOM at a dose of 10 mg/kg on day -5. On day 0, the mice were orally treated with 1.5% DSS for 4 days, followed by regular drinking water until day 21. The DSS treatment was repeated for two additional cycles. On day 56, the colon was removed for histological and biochemical analyses.

### Separation of colonic epithelial and non-epithelial cells

The colon was removed, opened longitudinally, washed with PBS, and incubated with PBS containing 5 mM EDTA with shaking for 30 min at 37 °C. The tissue was separated into CECs and non-CECs under a stereomicroscope, and the cells were washed with PBS before use.

### Microarray analysis

Total RNA extracted from mouse colons was purified using the RNeasy Mini Kit (Qiagen). The quality of the RNA was assessed with a 2100 Bioanalyzer (Agilent Technologies). cRNA targets were synthesized and hybridized with a Whole Mouse Genome Microarray in accordance with the manufacturer’s instructions (Agilent Technologies). The array slides were scanned using a SureScan Microarray Scanner (Agilent Technologies). Microarray data were analyzed with Agilent’s Feature Extraction Software. The GEO accession number for the microarray is GSE102389.

### Lipidomics analysis

ESI-MS was performed in accordance with our current protocol^55^. In brief, for detection of phospholipids, tissues were soaked in 10 volumes of 20 mM Tris-HCl (pH 7.4) and homogenized with a Polytron homogenizer. Lipids were extracted from the homogenates by the method of Bligh and Dyer^84^. MS analysis was performed using a 4000Q-TRAP quadrupole-linear ion trap hybrid mass spectrometer (AB Sciex) with liquid chromatography (LC; NexeraX2 system; Shimazu). The samples were applied to a Kinetex C18 column (1 × 150 mm i.d., 1.7 μm particle) (Phenomenex) coupled to ESI-MS/MS. The samples injected by an autosampler (10 μl) were separated by a step gradient with mobile phase A (acetonitrile/methanol/water = 1:1:1 [v/v/v] containing 5 μM phosphoric acid and 1 mM ammonium formate) and mobile phase B (2-propanol containing 5 μM phosphoric acid and 1 mM ammonium formate) at a flow rate of 0.2 ml/min at 50 °C. For detection of fatty acids and their oxygenated metabolites, tissues were soaked in 10 volumes of methanol and then homogenized with a Polytron homogenizer. After overnight incubation at −20 °C, water was added to the mixture to give a final methanol concentration of 10% (v/v). The samples in 10% methanol were applied to Oasis HLB cartridges (Waters), washed with 10 ml of hexane, eluted with 3 ml of methyl formate, dried under N_2_ gas, and dissolved in 60% methanol. The samples were then applied to a Kinetex C18 column (1 × 150 mm i.d., 1.7 μm particles) (Phenomenex) coupled to ESI-MS/MS as described above. The samples injected by an autosampler (10 μl) were separated using a step gradient with mobile phase C (water containing 0.1% acetic acid) and mobile phase D (acetonitrile/methanol = 4:1; v/v) at a flow rate of 0.2 ml/min at 45 °C. Identification was conducted using multiple reaction monitoring (MRM) transition and retention times, and quantification was performed based on the peak area of the MRM transition and the calibration curve obtained with an authentic standard for each compound. As internal standards, *d5*-labeled EPA and 17:0 LPC (1 nmol; Cayman Chemicals) were added to each sample.

### PLA_2_ enzyme assay using natural membranes

Total phospholipids were extracted from mouse colon and further purified by straight-phase chromatography. The samples extracted in chloroform were applied to a Sep-Pak Silica Cartridge (Waters), washed sequentially with acetone and chloroform/methanol (9/1; v/v), eluted with chloroform/methanol (3/1; v/v), and dried under N_2_ gas. The membrane mimic composed of tissue-extracted lipids (10 μM) was sonicated for 5 min in 100 mM Tris-HCl (pH 7.4) containing 4 mM CaCl_2_ and then incubated for appropriate periods with the mature form of recombinant human sPLA_2_-III protein^83^ (1–5 ng/ml) at 37 °C for 30 min. After incubation, the lipids were mixed with internal standards, extracted, and subjected to LC-MS for detection of fatty acids and lysophospholipids, as noted above.

### Statistical analysis

Data are expressed as mean ± SEM. Statistical significance of differences between groups was evaluated by two-tailed Student’s *t* test or one-way ANOVA at a significance level of *P* < 0.05.

## Electronic supplementary material

Supplemental information
