# Supplementary material for: Group III phospholipase A2 promotes colitis and colorectal cancer
Source: Sci Rep. 2017 Sep 25;7:12261. doi: 10.1038/s41598-017-12434-z (PMC5612992; doi:10.1038/s41598-017-12434-z)
Supplement: Supplementary file 1 — Supplemental information [file 41598_2017_12434_MOESM1_ESM.pdf]

Supplementary information

Group III phospholipase A_2 promotes colitis and colorectal cancer

Remi Murase[1,2], Yoshitaka Taketomi[1,2], Yoshimi Miki[1,2], Yasumasa Nishito[3], Moe Saito[2,4], Kiyoko Fukami[4], Kei Yamamoto[2,5,6], and Makoto Murakami[1,2,7]

[1]Laboratory of Microenvironmental and Metabolic Health Science, Center for Disease Biology and Integrative Medicine, Graduate School of Medicine, the University of Tokyo, 7-3-1 Hongo, Bunkyo-ku, Tokyo 113-8655, Japan.
[2]Lipid Metabolism Project, Tokyo Metropolitan Institute of Medical Science, 2-1-6 Kamikitazawa, Setagaya-ku, Tokyo 156-8506, Japan.
[3]Core Technology and Research Center, Tokyo Metropolitan Institute of Medical Science, 2-1-6 Kamikitazawa, Setagaya-ku, Tokyo 156-8506, Japan.
[4]Laboratory of Genome and Biosignal, Tokyo University of Pharmacy and Life Science, 1432-1 Horinouchi, Hachioji, 192-0392 Tokyo, Japan.
[5]Faculty of Bioscience and Bioindustry, Tokushima University, Tokushima 770-8513, Japan.
[6]PRIME, Japan Agency for Medical Research and Development, Tokyo 100-0004, Japan.
[7]AMED-CREST, Japan Agency for Medical Research and Development, Tokyo 100-0004, Japan.

Running title: Group III $sPLA_2$ and colorectal diseases

Address correspondence to: Makoto Murakami, Ph.D. at the Laboratory of Microenvironmental and Metabolic Health Sciences, Center for Disease Biology and Integrative Medicine, Graduate School of Medicine, the University of Tokyo, 7-3-1 Hongo, Bunkyo-ku, Tokyo 113-0033, Japan.
Tel: 81-3-5841-1431; Fax: 81-3-5841-1434; Email: makmurak@m.u-tokyo.ac.jp

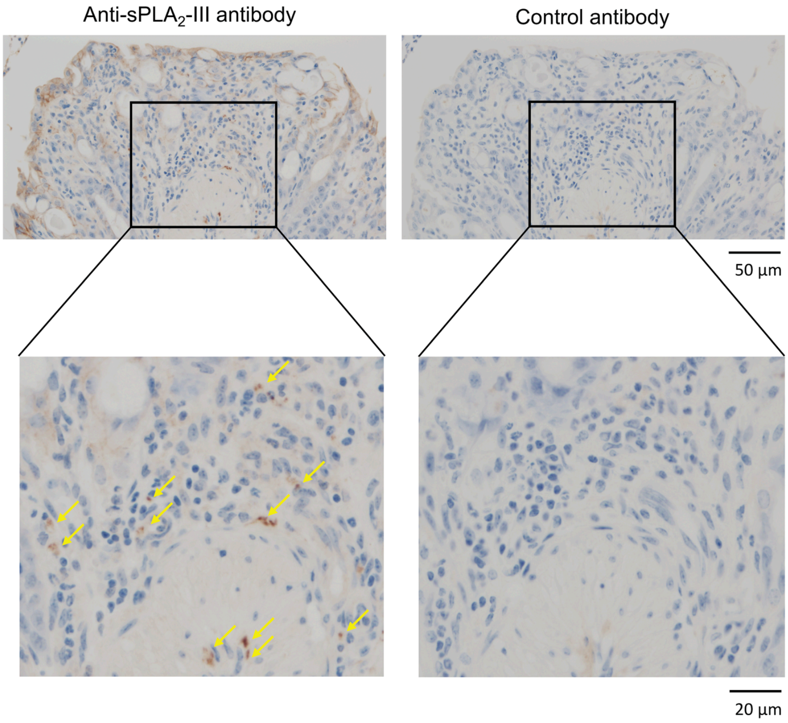

Supplementary Figure 1.

Immunohistochemisty of sPLA$_2$-III in the colon of DSS-treated mice, related to Fig. 3.

The colon of WT mice treated for 6 days with DSS was subjected to immunohistochemistry using anti-sPLA$_2$-III or control antibody. The anti-sPLA$_2$-III antibody mainly stained collapsing epithelial cells. Although most inflammatory cells infiltrating beneath the damaged epithelium were sPLA$_2$-III-negative, a minor population of immune cells was sporadically stained for sPLA$_2$-III (*arrows*). Control antibody gave no staining.

Supplementary Table 1.

Microarray gene profiling of lipid-metabolic genes in the colons of *Pla2g3*$^{+/+}$ and *Pla2g3*$^{-/-}$ mice, related to Fig. 2e.

Expression profiles of genes associated with lipid biosynthesis, degradation, and consumption in *Pla2g3*$^{-/-}$ (KO) colon relative to littermate *Pla2g3*$^{+/+}$ (WT) colon with or without AOM treatment, as assessed by DNA microarray analysis. Fold changes (KO/WT) are indicated. Equal amounts of total RNA pooled from four mice for each genotype were used. Genes showing a >2-fold increase (red) or >50% reduction (blue) in expression are highlighted.

Lipogenesis and lipid mediator synthesis

Gene Name	Description	Accession No.	Normal KO/WT	AOM KO/WT
Acss1	Acyl-CoA synthetase short-chain family member 1	NM_080575	0.88	2.16
Acss2	Acyl-CoA synthetase short-chain family member 2	NM_019811	0.87	5.61
Acsm3	Acyl-CoA synthetase medium-chain family member 3	NM_212441	0.98	6.84
Acsl1	Acyl-CoA synthetase long-chain family member 1	NM_007981	0.92	1.48
Acsl3	Acyl-CoA synthetase long-chain family member 3	NM_028817	0.98	2.85
Acsl4	Acyl-CoA synthetase long-chain family member 4	NM_207625	0.78	0.38
Acsl5	Acyl-CoA synthetase long-chain family member 5	NM_027976	1.13	1.01
Acsf2	Acyl-CoA synthetase family member 2	NM_153807	0.99	1.22
Acsf3	Acyl-CoA synthetase family member 3	NM_144932	1.06	1.45
Elovl1	Elongation of very long chain fatty acids family member 1	NM_001039176	1.04	1.03
Elovl5	Elongation of very long chain fatty acids family member 5	NM_134255	0.82	0.75
Elovl6	Elongation of very long chain fatty acids family member 6	NM_130450	1.15	0.54
Elovl7	Elongation of very long chain fatty acids family member 7	NM_029001	1.00	1.65
Fads1	Fatty acid desaturase 1	NM_146094	0.94	0.29
Fads2	Fatty acid desaturase 2	NM_019699	0.86	1.03
Fads3	Fatty acid desaturase 3	NM_021890	1.01	1.09
Fasn	Fatty acid synthase	NM_007988	0.64	0.68
Ptgs1	Prostaglandin-endoperoxide synthase 1 (COX-1)	NM_008969	0.89	0.95
Ptgs2	Prostaglandin-endoperoxide synthase 2 (COX-2)	NM_011198	0.73	0.08
Ptges	Prostaglandin E synthase	NM_022415	0.98	0.24
Ptges2	Prostaglandin E synthase 2	NM_133783	1.10	1.11
Ptges3	Prostaglandin E synthase 3 (cytosolic)	NM_019766	1.15	0.66
Ptgis	Prostaglandin I_2 (prostacyclin) synthase	NM_008968	0.95	1.15
Tbxas1	Thromboxane A synthase 1, platelet	NM_011539	0.84	0.26
Aloxe3	Arachidonate lipoxygenase 3	NM_011786	0.23	1.00
Alox5	Arachidonate 5-lipoxygenase	NM_009662	1.03	0.76
Alox12	Arachidonate 12-lipoxygenase	NM_007440	1.08	0.02
Alox12b	Arachidonate 12-lipoxygenase, 12R type	NM_009659	0.84	1.32
Alox12e	Arachidonate lipoxygenase, epidermal	NM_145684	0.85	0.29

Agpat1	1-acylglycerol-3-phosphate O-acyltransferase 1	NM_001163379	1.00	2.28
Agpat2	1-acylglycerol-3-phosphate O-acyltransferase 2	NM_026212	1.16	0.78
Agpat3	1-acylglycerol-3-phosphate O-acyltransferase 3	NM_053014	0.94	0.67
Agpat4	1-acylglycerol-3-phosphate O-acyltransferase 4	NM_026644	1.01	6.17
Agpat5	1-acylglycerol-3-phosphate O-acyltransferase 5	NM_026792	0.95	1.01
Agpat6	1-acylglycerol-3-phosphate O-acyltransferase 6	NM_018743	1.03	0.86
Agpat9	1-acylglycerol-3-phosphate O-acyltransferase 9	NM_172715	1.11	1.06
Lpcat1	Lysophosphatidylcholine acyltransferase 1	NM_145376	0.93	1.65
Lpcat2	Lysophosphatidylcholine acyltransferase 2	NM_173014	5.19	2.74
Lpcat3	Lysophosphatidylcholine acyltransferase 3	NM_145130	1.05	0.76

Phospholipase A_2s and related enzymes

Gene Name	Description	Accession No.	Normal KO/WT	AOM KO/WT
Pla1a	Phospholipase A_1 member A	NM_134102	0.80	0.28
Pla2g2c	Phospholipase A_2, group IIC (sPLA$_2$-IIC)	NM_008868	1.23	3.49
Pla2g2d	Phospholipase A_2, group IID (sPLA$_2$-IID)	NM_011109	0.61	0.04
Pla2g2e	Phospholipase A_2, group IIE (sPLA$_2$-IIE)	NM_012044	0.77	0.21
Pla2g2f	Phospholipase A_2, group IIF (sPLA$_2$-IIF)	NM_012045	0.88	1.11
Pla2g5	Phospholipase A_2, group V (sPLA$_2$-V)	NM_011110	0.85	2.92
Pla2g10	Phospholipase A_2, group X (sPLA$_2$-X)	NM_001291009	0.90	3.09
Pla2g12a	Phospholipase A_2, group XIIA (sPLA$_2$-XIIA)	NM_183423	0.97	0.19
Pla2g12b	Phospholipase A_2, group XIIB (sPLA$_2$-XIIB)	NM_023530	1.04	3.80
Pla2g4a	Phospholipase A_2, group IVA (cPLA$_2\alpha$)	NM_008869	0.99	1.53
Pla2g4b	Phospholipase A_2, group IVB (cPLA$_2\beta$)	NM_145378	1.01	1.18
Pla2g4f	Phospholipase A_2, group IVF (cPLA$_2\zeta$)	NM_001024145	1.10	6.06
Pnpla2	Patatin-like phospholipase domain containing 2 (adipose triglyceride lipase)	NM_001163689	0.95	1.37
Pnpla6	Patatin-like phospholipase domain containing 6 (iPLA$_2\delta$)	NM_001122818	1.14	1.02
Pnpla7	Patatin-like phospholipase domain containing 7	NM_146251	0.97	0.80
Pnpla8	Patatin-like phospholipase domain containing 8 (iPLA$_2\gamma$)	NM_026164	1.15	1.44
Pla2g6	Phospholipase A_2, group VI (iPLA$_2\beta$)	NM_001199023	1.13	0.42
Pla2g7	Phospholipase A_2, group VII (platelet-activating factor acetylhydrolase, plasma)	NM_013737	0.62	0.26
Pafah2	Platelet-activating factor acetylhydrolase 2	NM_001285872	1.03	2.10
Prdx6	Peroxiredoxin 6	NM_007453	1.19	4.41
Pla2g15	Phospholipase A_2, group XV	NM_133792	0.90	0.41
Pla2g16	Phospholipase A_2, group XVI	NM_139269	0.98	1.15
Lypla1	Lysophospholipase 1	NM_008866	1.09	1.47
Lypla2	Lysophospholipase 2	NM_011942	0.98	1.13
Abhd1	Abhydrolase domain containing 1	NM_021304	0.99	2.78
Abhd2	Abhydrolase domain containing 2	NM_018811	1.08	1.44
Abhd4	Abhydrolase domain containing 4	NM_134076	1.22	0.66
Abhd5	Abhydrolase domain containing 5	NM_026179	1.26	1.24

Abhd6	Abhydrolase domain containing 6	NM_025341	1.49	1.51
Abhd8	Abhydrolase domain containing 8	NM_022419	0.93	0.67
Abhd10	Abhydrolase domain containing 10	NM_172511	1.05	0.89
Abhd11	Abhydrolase domain containing 11	NM_145215	0.93	1.19
Abhd12	Abhydrolase domain containing 12	NM_024465	0.93	0.29
Abhd13	Abhydrolase domain containing 13	NM_026868	2.45	1.19
Abhd14a	Abhydrolase domain containing 14A	NM_001110271	1.07	0.68
Abhd14b	Abhydrolase domain containing 14B	NM_029631	1.00	2.66
Abhd15	Abhydrolase domain containing 15	NM_026185	1.19	0.28
Abhd16a	Abhydrolase domain containing 16A	NM_178592	1.00	1.39
Abhd17a	Abhydrolase domain containing 17A	NM_145421	1.01	1.09
Abhd17b	Abhydrolase domain containing 17B	NM_146096	0.91	1.48

Other lipases

Gene Name	Description	Accession No.	Normal KO/WT	AOM KO/WT
Plbd1	Phospholipase B domain containing 1	NM_025806	1.05	3.07
Plbd2	Phospholipase B domain containing 2	NM_023625	1.02	1.04
Plcb1	Phospholipase C, beta 1	NM_001145830	0.52	1.73
Plcb3	Phospholipase C, beta 3	NM_001290349	0.99	2.97
Plcb4	Phospholipase C, beta 4	NM_013829	1.06	3.12
Plcg1	Phospholipase C, gamma 1	NM_021280	1.23	0.28
Plcg2	Phospholipase C, gamma 2	NM_172285	0.84	0.29
Plcd1	Phospholipase C, delta 1	NM_019676	1.08	4.25
Plcd3	Phospholipase C, delta 3	NM_152813	1.35	0.57
Plce1	Phospholipase C, epsilon 1	NM_019588	1.03	4.16
Plch1	Phospholipase C, eta 1	NM_001177732	0.86	1.28
Plch2	Phospholipase C, eta 2	NM_175556	1.06	4.22
Pld1	Phospholipase D1	NM_001164056	1.10	2.18
Pld2	Phospholipase D2	NM_008876	0.95	0.46
Pld3	Phospholipase D family, member 3	NM_011116	0.97	0.47
Pld4	Phospholipase D family, member 4	NM_178911	0.95	0.41
Pld6	Phospholipase D family, member 6	NM_001290283	1.10	1.04
Lpl	Lipoprotein lipase	NM_008509	1.41	0.51
Lipg	Lipase, endothelial	NM_010720	1.24	10.19
Liph	Lipase, member H	NM_153404	1.04	2.22
Lipo1	Lipase, member O1	NM_001013770	1.01	1.16
Mgll	Monoglyceride lipase	NM_001166251	1.14	3.27
Dagla	Diacylglycerol lipase, alpha	NM_198114	0.96	2.35
Daglb	Diacylglycerol lipase, beta	NM_144915	0.95	1.89
Enpp1	Ectonucleotide pyrophosphatase/phosphodiesterase 1	NM_008813	0.88	1.17

Fatty acid β-oxidation

Gene Name	Description	Accession No.	Normal KO/WT	AOM KO/WT
Acad8	Acyl-CoA dehydrogenase family, member 8	NM_025862	0.97	2.19
Acad9	Acyl-CoA dehydrogenase family, member 9	NM_172678	0.96	1.11
Acad10	Acyl-CoA dehydrogenase family, member 10	NM_028037	1.03	1.10
Acad11	Acyl-CoA dehydrogenase family, member 11	NM_175324	1.21	3.07
Acad12	Acyl-CoA dehydrogenase family, member 12	NM_178799	1.32	1.57
Acads	Acyl-CoA dehydrogenase, short chain	NM_007383	0.97	2.13
Acadm	Acyl-CoA dehydrogenase, medium chain	NM_007382	1.00	3.48
Acadl	Acyl-CoA dehydrogenase, long chain	NM_007381	1.00	1.32
Ech1	Enoyl CoA hydratase 1, peroxisomal	NM_016772	1.00	1.86
Echs1	Enoyl CoA hydratase, short chain, 1, mitochondrial	NM_053119	0.99	1.96
Echdc1	Enoyl CoA hydratase domain containing 1	NM_025855	0.98	1.02
Echdc2	Enoyl CoA hydratase domain containing 2	NM_026728	1.11	0.15
Echdc3	Enoyl CoA hydratase domain containing 3	NM_024208	1.02	1.96
Ehhadh	Enoyl-CoA, hydratase/3-hydroxyacyl CoA dehydrogenase	NM_023737	1.10	4.53
Hadha	Hydroxyacyl-CoA dehydrogenase/3-ketoacyl-CoA thiolase/enoyl-CoA hydratase (trifunctional protein), alpha subunit	NM_178878	1.07	1.32
Acat1	Acetyl-CoA acetyltransferase 1	NM_144784	1.09	2.51
Acat2	Acetyl-CoA acetyltransferase 2	NM_009338	0.79	1.11
Acat3	Acetyl-CoA acetyltransferase 3	NM_153151	0.79	0.80

Supplementary Table 2.
Primers and probes for quantitative RT-PCR.

Name	Assay No. (Applied Biosystems)
Pla2g3	Mm01191142_m1
Il1b	Mm00434228_m1
Il6	Mm00446190_m1
Il17a	Mm00439618_m1
Il22	Mm00444241_m1
Il23	Mm01160011_g1
Tnf	Mm00443258_m1
Ptgs2	Mm00478374_m1
Ptges	Mm00452105_m1
Mcpt1	Mm00656886_g1
Mcpt2	Mm00484932_m1
Arg1	Mm00475988_m1
Chil3 (Ym1)	Mm00657889_mH
Foxp3	Mm00475162_m1
Vegfa	Mm01281449_m1
Mmp9	Mm00442991_m1
Cldn1	Mm00516701_m1
Muc2	Mm01276696_m1
